# Supplementary material for: Chromatic Bacteria – A Broad Host-Range Plasmid and Chromosomal Insertion Toolbox for Fluorescent Protein Expression in Bacteria
Source: Front Microbiol. 2018 Dec 12;9:3052. doi: 10.3389/fmicb.2018.03052 (PMC6315172; doi:10.3389/fmicb.2018.03052)
Supplement: Supplementary file 1 [file Data_Sheet_1.docx]

Supplementary Material

Chromatic bacteria – A broad host-range plasmid and chromosomal insertion toolbox for fluorescent protein expression in bacteria

Rudolf O. Schlechter^1,2^, Hyunwoo Jun^1#^, Michał Bernach^1,2#^, Simisola Oso^1^, Erica Boyd^1^, Dian A. Muñoz-Lintz^1^, Renwick C. J. Dobson^1,2,3^, Daniela M. Remus^1,2,4^, and Mitja N. P. Remus-Emsermann^1,2*^

^1^School of Biological Sciences, University of Canterbury, Christchurch, New Zealand.

^2^Biomolecular Interaction Centre, University of Canterbury, Christchurch, New Zealand.

^3^Bio21 Molecular Science and Biotechnology Institute, Department of Biochemistry and Molecular Biology, University of Melbourne, Parkville, Victoria 3010, Australia.

^4^Protein Science & Engineering, Callaghan Innovation, School of Biological Sciences, University of Canterbury, Christchurch, New Zealand.

^#^ Both authors contributed equally to this work

*** Correspondence:**Mitja N. P. Remus-Emsermann
mitja.remus-emsermann@canterbury.ac.nz

# Supplementary Figures


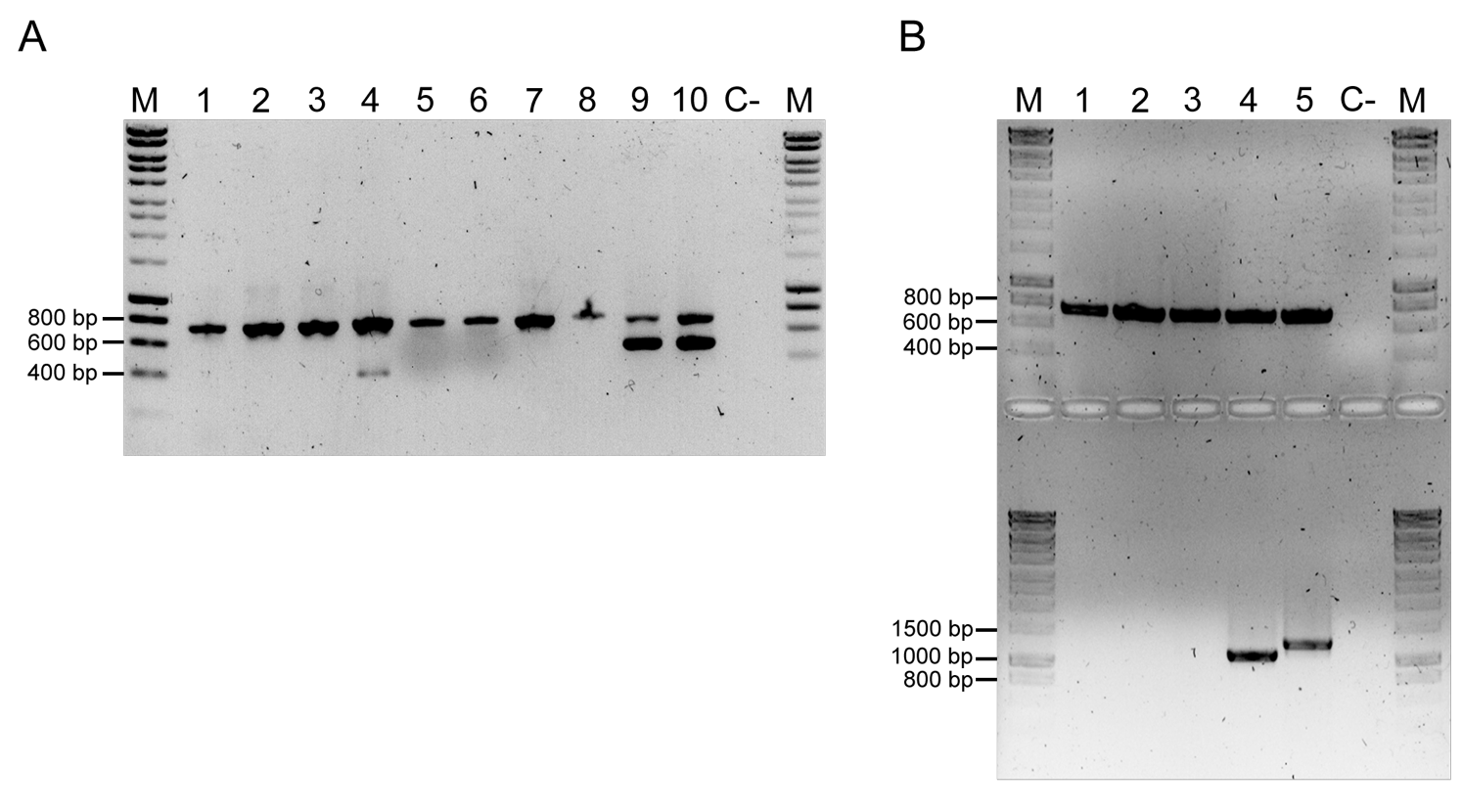


**Supplementary Figure 1.** Validation of transposon integration into recipient strains by PCR. (A) Tn*5* insertions. Amplification of inserts and pMRE-Tn5 plasmid backbone yields fragments of 723 bp and 503 bp, respectively. 1: *Bradyrhizobium* sp. Leaf396::MRE-Tn5-165; 2: *Methylobacterium* sp. Leaf92::MRE-Tn5-165; 3: *Sphingomonas melonis* FR1::MRE-Tn5-145; 4: *Sphingomonas phyllosphaerae* FA2::MRE-Tn5-145; 5: *Erwinia amylovora* CFBP1430S::MRE-Tn5-145; 6: *Pantoea agglomerans* 299R::MRE-Tn5-145; 7: *Pseudomonas citronellolis* P3B5::MRE-Tn5-145; 8: *Pseudomonas syringae* pv. *syringae* B728a::MRE-Tn5-145; 9: *Escherichia coli* S17-1 (pMRE-Tn5-145). 10: pMRE-Tn5-145. (B) Tn*7* insertions. Amplification of insert (upper gel) and pMRE-Tn7 plasmid backbone (lower gel) yields fragments of around 723 bp and either 732 bp or 1086 bp, depending on the orientation of the insert in the plasmid. 1: *S. melonis* FR1::MRE-Tn7-145; 2: *E. amylovora* CFBP1430S::MRE-Tn7-145; 3: *P. agglomerans* 299R::MRE-Tn7-145; 4: *E. coli* S17-1 (pMRE-Tn7-145). 5: pMRE-Tn7-145. In all cases, M: HyperLadder^TM^ 1kb DNA marker, C-: Negative control, no DNA added.

**
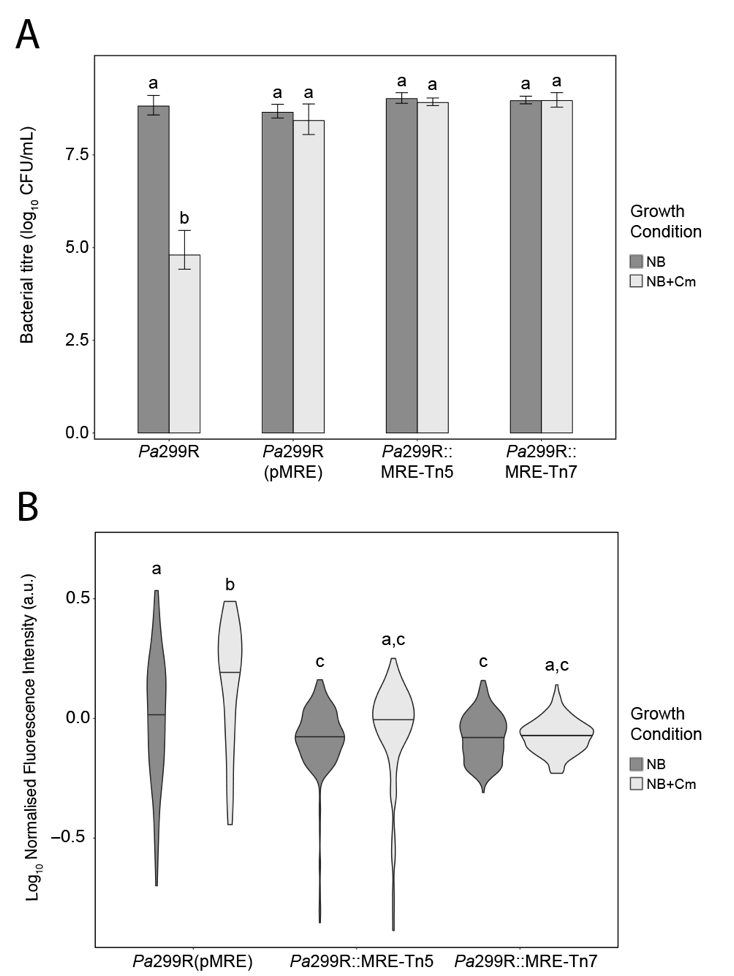
**

**Supplementary Figure 2.** Effect of antibiotic pressure in growth and fluorescence of *Pantoea agglomerans* 299R (*Pa*299R) harbouring either a pMRE plasmid, a Tn*5* insertion, or a Tn*7* insertion. (A) Colony forming units from overnight cultures of *Pa*299R, *Pa*299R (pMRE135), *Pa*299R::MRE-Tn5-145, and *Pa*299R::MRE-Tn7-145 growing in nutrient broth (NB) with or without chloramphenicol (Cm). (B) Single-cell fluorescence intensity from overnight cultures of *Pa*299R, *Pa*299R (pMRE135), *Pa*299R::MRE-Tn5-145, and *Pa*299R::MRE-Tn7-145 growing in NB with or without Cm. Fluorescence intensity of single cells was normalised by exposure time. a. u. = arbitrary units. Letters indicate statistical differences between the mean of each condition (*p* < 0.001, two-way ANOVA).
